# The EMCV protein 2B* is required for efficient cell lysis via both caspase-3-dependent and -independent pathways during infection

**DOI:** 10.1099/jgv.0.002075

**Published:** 2025-02-10

**Authors:** Samantha K. Nguyen, Edward Long, James R. Edgar, Andrew E. Firth, Hazel Stewart

**Affiliations:** 1 Department of Pathology, University of Cambridge, Cambridge, UK

**Keywords:** apoptosis, caspase-3, cell death, encephalomyocarditis virus, picornavirus, pyroptosis

## Abstract

2B* is a poorly characterized protein encoded by an overlapping ORF in the genome of encephalomyocarditis virus (EMCV). We have previously found 2B* to have a role in innate immune antagonism; however, this role is distinct from an earlier described phenotype whereby 2B*KO viruses exhibit extremely small plaques compared to WT. Here, we report that the small plaque phenotype is recapitulated by novel EMCV mutant viruses harbouring mutations across the C-terminal domain of 2B*, confirming a functional role of 2B* in promoting viral spread. We found that 2B*KO EMCV displays impaired extracellular virus titres compared to WT EMCV, despite producing a similar number of infectious particles overall. This correlates with a reduction in cell lysis and lower levels of caspase-3 cleavage occurring during infection. Further investigation using caspase inhibitors and knockout cells revealed that WT EMCV can utilize both caspase-3-dependent and caspase-3-independent pathways to achieve cell lysis, the former of which is likely to be GSDME-mediated pyroptosis. 2B* increases the efficiency of both lytic pathways through an as-yet-undefined mechanism. This work reveals 2B*, a protein only found in EMCV, to be a key regulator of multiple lytic cell death pathways, leading to enhanced rates of virus release. This explains the rapid cell death observed during WT EMCV infection and the small plaque phenotype seen in both 2B*KO and previously described 2B* mutant viruses.

## Data Availability

**Fig. 1.**
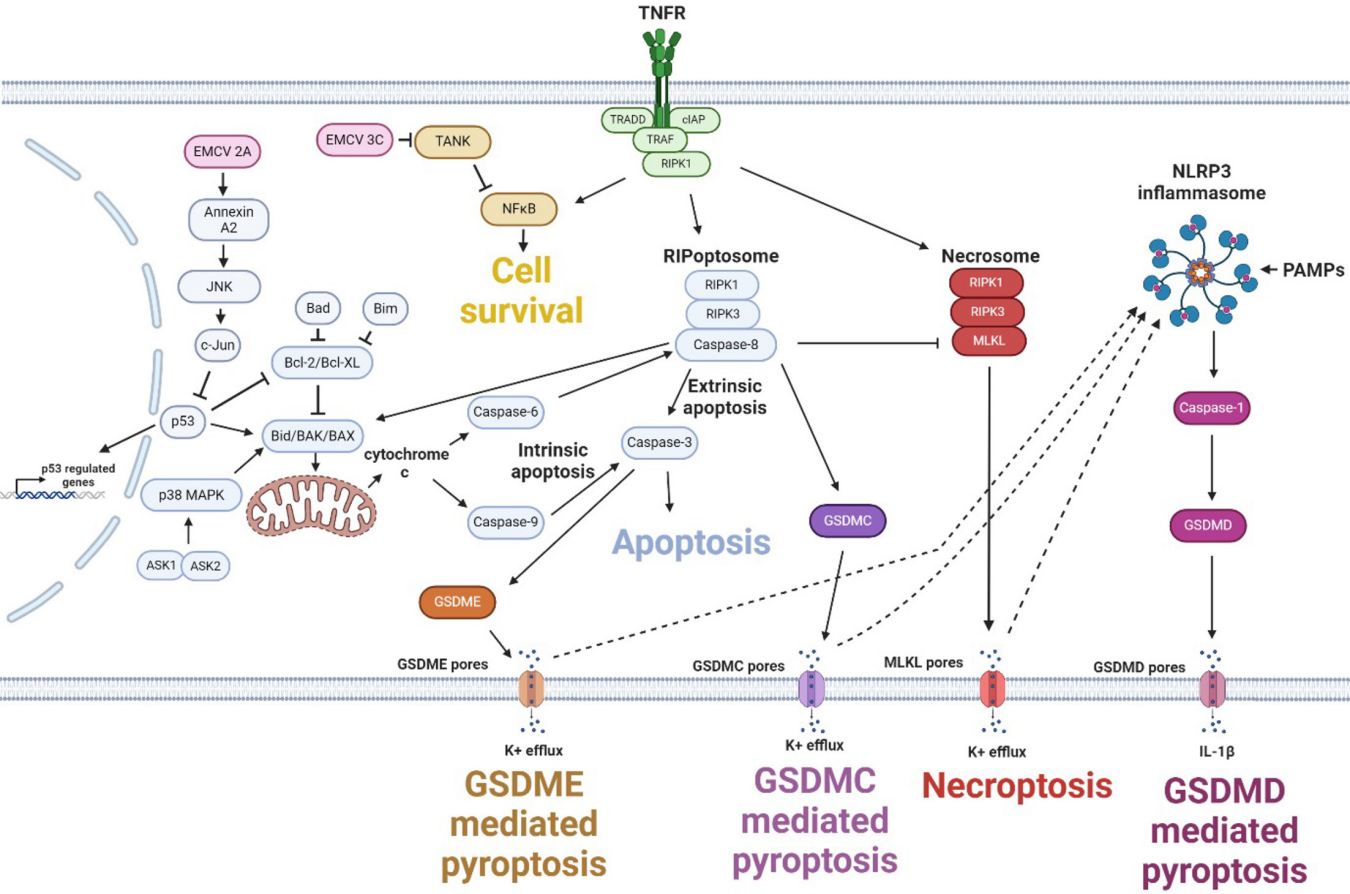
The interlinked cell death pathways of apoptosis, pyroptosis and necroptosis. Oligomerized GSDMC, GSDMD, GSDME and MLKL form non-selective pores, allowing the release of all cellular contents, including the indicated ions and immune signalling molecules, leading to the lytic cell death pathways of pyroptosis or necroptosis, as indicated. The resulting K^+^ efflux is also capable of activating the NLRP3 inflammasome in macrophage cells, as indicated by the dashed arrows. Created in BioRender. Stewart, H. (2025) https://BioRender.com/o62m219.

The following accession numbers are described in this study and are available on GenBank: NM_004403.3 [*Homo sapiens* gasdermin E (GSDME), transcript variant 1], DQ294633.1 (EMCV subtype mengovirus cDNA, pMC0), X56019.1 (TMEV) and XM_005087183.4 (hamster GSDME mRNA). The accession numbers used to generate Fig. 1 are listed therein.

## Introduction

Encephalomyocarditis virus (EMCV, species *Cardiovirus rueckerti*, family *Picornaviridae*) (previously known as Cardiovirus A) is capable of infecting a wide range of mammalian hosts, causing encephalitis, diabetes and myocarditis in mice and primates as well as reproductive disorders in pigs [1]. Despite the wide tissue and host tropism, it is primarily considered a rodent virus and is an important model picornavirus for studying host–pathogen interactions. The genome encodes a polyprotein, which is cleaved into 12 proteins: a leader protein (L) followed by the capsid proteins (1ABCD, also known as VP4, VP2, VP3 and VP1) and non-structural proteins (2ABC and 3ABCD). Cleavage is performed by the viral protease, 3C^pro^, with the exception of 2A and 2B, which are separated co-translationally by the StopGo mechanism [2,3].

In 2011, the genome of EMCV was found to encode a thirteenth protein, 2B*, in an overlapping ORF within the 2B coding sequence. 2B* was originally identified by the presence of increased synonymous site conservation in this region accompanied by the absence of −1 frame stop codons in all EMCV sequences available [4]. 2B* is translated via programmed ribosomal frameshifting (PRF), stimulated by the viral 2A protein binding to an RNA pseudoknot beginning eight nt downstream of the frameshift site [4,6]. Unusually, due to the transactivation by viral protein 2A, the PRF is temporally regulated, increasing in efficiency from 0% at early stages of infection to 70% by 6–8 h post-infection (hpi), thus resulting in time-dependent translation of 2B* [6]. Both knockout and truncation mutations of 2B* result in a small plaque phenotype, indicative of a reduction in viral spread [4,6, 7]. However, the mechanism by which 2B* affects viral spread is undetermined. We recently found that EMCV 2B* interacts with the 14-3-3 family of host proteins via a conserved C-terminal motif, resulting in reduced activation of the innate immune response [7]. As ablating the 2B*:14-3-3 interaction was not found to reduce plaque size, it appears that these two functions are unrelated and 2B* is a multi-functional viral accessory protein, essential for efficient viral spread.

The *Cardiovirus* genus includes six species, several of which have only one or a very few known isolates [8]. Multiple isolates have however been found for EMCV and Theiler’s murine encephalomyelitis virus (TMEV) (*Cardiovirus theileri*) [9]. Despite their close relationship, only the EMCV species of cardioviruses encodes a lengthy 2B* polypeptide. Although frameshifting was confirmed to occur at the same site in TMEV, here, it results in the production of a peptide of only 14 residues in length. PRF in TMEV therefore temporally regulates the ratio of structural to non-structural protein synthesis but does not appear to produce an additional functional protein [10,12].

The broad range of pathogenicities caused by EMCV is considered to be partly due to the rapid cell lysis induced during infection of multiple cell lines [1,13, 14], to the point where specifically targeted EMCV infection has been considered as an oncolytic therapeutic [15,16]. The lytic viral release is highly efficient and induces a severe inflammatory response, causing acute clinical symptoms. Viral propagation is further heightened by the ability of EMCV proteins L [17,18], 3C^pro^ [19,21], 2C [22,23] and VP3 [24] to antagonize the host innate immune system and reduce antiviral responses; 2B* may now also be added to this list [7].

**Fig. 2.**
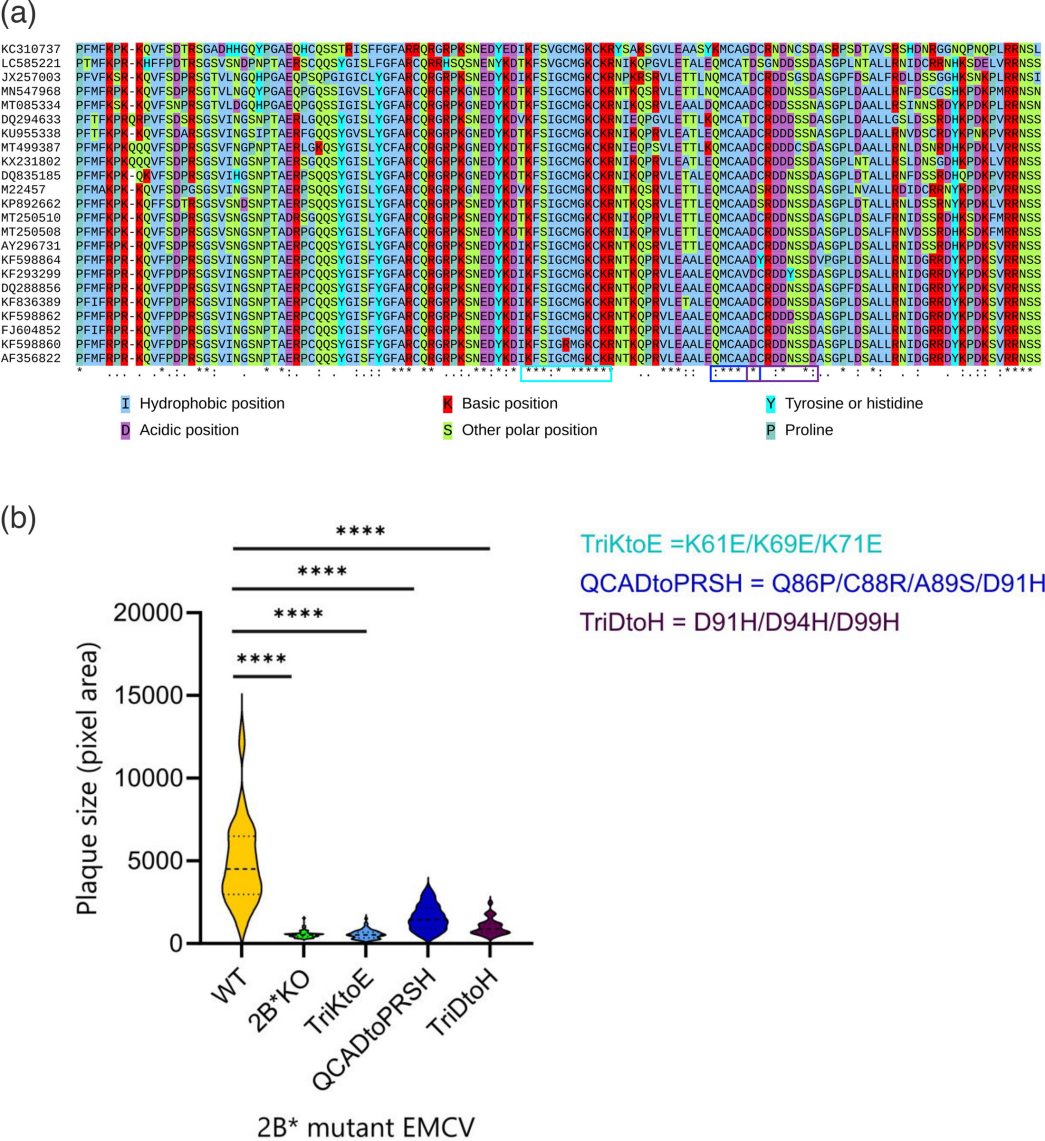
2B* mutant viruses reproduce the small plaque phenotype associated with 2B*KO EMCV. (**a**) Alignment of all unique *C. rueckerti* (Cardiovirus A) 2B* sequences available on GenBank (3 October 2022). Duplicate sequences have been discarded. aa are colour coded according to their physicochemical properties. Asterisks indicate completely conserved residues, whereas colons and dots indicate stronger and weaker conservation (based on identity and shared physicochemical properties). Regions targeted by mutation are boxed in cyan, blue or purple. (**b**) BSR cells were infected with the respective viruses for 1 h prior to being overlaid with a semi-solid medium (1% low melting point agarose) and incubated for 48 h. Data shown are the size distribution of 60 randomly chosen plaques sampled over three biological repeats. Dotted lines indicate the median, upper and lower quartiles of the plaque size for each mutant virus. Statistical analysis (ratio-paired t-test): ****P≤0.0001.

The mechanism by which EMCV induces cell lysis is poorly understood. Lytic cell death is essentially due to the formation of pores in the cell membrane, leading to cytosolic leakage and eventual membrane disintegration (in direct contrast to apoptotic cell death, see below). The most common mechanisms of lytic death can be broadly categorized into either necroptosis or pyroptosis, based on the components of the pore complex: necroptosis occurs when RIPK1 and RIPK3 are activated and phosphorylate MLKL, which oligomerizes to form pores [25], whereas pyroptosis is mediated by the cell death executor protein family of gasdermins (GSDMA to GSDME), which form pores upon cleavage [26] (Fig. 2).

In comparison with cell lysis, apoptosis (an essential host defence mechanism against viral infection) [27,29] results in cell death and fragmentation into membrane-bound apoptotic bodies. Apoptosis is tightly regulated and can be induced by a variety of signals, which activate two distinct pathways: intrinsic and extrinsic apoptosis. Both pathways converge at the point of caspase-3 cleavage and activation. As all cellular contents remain tightly contained within membranes, until the removal of the apoptotic bodies by phagocytic cells, apoptosis leads to viral entrapment, preventing viral spread. This viral entrapment means that plaque size is often limited by apoptosis [30]. One possibility therefore is that 2B* is an anti-apoptotic protein and 2B*KO EMCV-infected cells are undergoing apoptosis, rather than the canonical lytic cell death pathway induced by WT EMCV.

Currently, EMCV L, 2A and 3C^pro^ are known to have anti-apoptotic functions [21,30,33]. Despite the need to evade apoptosis, many picornaviruses activate this pathway but prevent the execution of the complete cascade. The purpose of the activation was perplexing until cleaved caspase-3, previously thought of as the hallmark of apoptosis, was identified as the source of GSDME-mediated pyroptosis [34,35]. The gasdermins are cleaved into their active forms by different proteases: GSDMA is cleaved by caspase-1 in non-mammals (whereas in mammals, the only known effectors are non-host proteins [36,38]); GSDMB by granzyme A [39]; GSDMC by caspase-6 and caspase-8 [40,41]; GSDMD by caspase-1, caspase-11 (in mice) or caspase-8 [42,43]; and GSDME by caspase-3 [34,35]. In all cases, cleavage of the full-length gasdermin leads to oligomerization of the N-terminal fragment, which inserts into the membrane in a pore-like formation and allows the release of cellular contents. Cleaved caspase-3 is now known to lead to GSDME-mediated pyroptosis during infection with picornaviruses EV71, foot and mouth disease virus (FMDV) or EMCV [34,44, 45].

The transactivation of PRF by 2A, leading to high levels of 2B* translation, means that 2B* may perform a function previously attributed to 2A, as deletion or many mutations of 2A will also prevent 2B* production. Additionally, as 2B* translation increases from 0 to 70% between 2 and 6 hpi [6], the mechanism by which 2B* contributes to plaque size is presumably limited to the later stages of infection. Given that plaque size, in the absence of a difference in viral titre, indicates the relative efficiency of viral egress, this study aimed to elucidate whether 2B* possessed an anti-apoptotic function or if it enhanced viral egress and hence determine how this intriguing protein influences the cell death pathway of EMCV-infected cells.

One-step virus growth curves and cytotoxicity assays indicated that 2B* temporally regulates lytic virus release. A combination of flow cytometry, live-cell imaging and immunodetection of cleaved cell death proteins identified an increase in caspase-3 activation and GSDME cleavage in WT EMCV-infected cells compared to infection with 2B*KO EMCV. We found that, rather than inhibiting apoptosis, 2B* appears to support lytic cell death. Experiments with caspase-3 knockout (KO) cell lines confirmed that EMCV-induced rapid cell lysis is supported by caspase-3 but also showed that WT EMCV can induce a caspase-3-independent lysis pathway, which 2B*KO EMCV cannot.

Together, these results indicate that the timed production of 2B* enables temporal regulation of lytic virus release via both a caspase-3-mediated cell lysis pathway, likely to be GSDME-mediated pyroptosis, and a second caspase-3-independent pathway. The result of this synergistic relationship between 2B* and caspase-3-mediated pyroptosis is that EMCV can induce cell death independently of both these factors, yet when both are present, lysis occurs more rapidly.

## Methods

### Multiple sequence alignment

*C. rueckerti* sequences (Cardiovirus A) were identified by searching the NCBI nr/nt database on 3 October 2022 with tblastn [46] using the NC_001479 polyprotein sequence as a query, giving 57 sequences with complete coverage of the 2B* region. The 2B* aa sequences were obtained, and duplicate 2B* sequences were discarded, leaving 23 unique representative 2B* sequences. These were aligned with muscle [47].

### Mammalian cell culture

BHK-21 (CCL-10, ATCC) (baby hamster kidney fibroblast), BSR (single-cell clone of BHK-21 cells), immortalized mouse embryonic fibroblast (MEF), murine macrophage RAW264.7 (RRID: CVCL_0493) and HEK293T (human embryonic kidney, CRL-3216, ATCC) cells were maintained in Dulbecco’s Modified Eagle Medium (DMEM) with high glucose (Sigma), supplemented with 10% (v/v) heat-inactivated FCS, 25 mM HEPES, 2 mM l-glutamine and non-essential aa (Sigma) (‘complete DMEM’). Caspase-3 KO HeLa cells [48] in addition to the parental cells (WT HeLa, human adenocarcinoma) were a kind gift from Dr Maddalena Nano and Professor Denise Montell (University of California, Santa Barbara). All cells were maintained in a humidified 5% CO_2_ atmosphere at 37 °C. All cell lines were confirmed to be mycoplasma free at regular intervals (MycoAlert™ PLUS Assay, Lonza).

### Cloning and expression plasmids

Viral clones containing mutations in conserved sites of 2B* (designated as TriKtoE 2B* EMCV, TriDtoH 2B* EMCV and QCADtoPRSH 2B*EMCV) were engineered via two-step overlap extension PCR. The desired amplicons were cloned into the WT EMCV cDNA clone with either *Bgl*II (Thermo Fisher Scientific FastDigest) and *Afl*II (Thermo Fisher Scientific FastDigest) or *Afl*II (Thermo Fisher Scientific FastDigest) and *Pas*I (Thermo Fisher Scientific) restriction enzymes. The entire genome of all mutant and WT clones was confirmed by Sanger sequencing (University of Cambridge Department of Biochemistry Sequencing Facility). To generate pcDNA3.1-myc-GSDME-FLAG, PCR was used to add an N-terminal myc tag to *Homo sapiens* gasdermin E (GSDME), transcript variant 1 (GenBank accession: NM_004403.3), already cloned into the pcDNA3.1-C-(k)DYK vector (GenScript, Hu25942). The amplicon was cloned into the original vector with *Bam*HI (Thermo Fisher Scientific FastDigest) and *Apa*I (Thermo Fisher Scientific FastDigest) restriction enzymes. The uncleavable GSDME mutant (pcDNA3.1-myc-GSDME-FLAG-D270A) was made using two-step overlap extension PCR.

### DNA transfection

HEK293T cells were transfected at 60–70% confluency in 6-well plates, having been seeded the day prior. 1.2 µg DNA and 3.6 µl TransIT were added to 120 µl Opti-MEM (Gibco) and incubated for 20 min. The media on the cells were changed to 2% FCS DMEM, and the transfection medium was added directly on top. The plates were rocked gently to mix and incubated at 37 °C in 5% CO_2_ for 24 h.

### Reverse genetics

The parental (WT) EMCV sequence has been previously described [6] and is based on the EMCV subtype mengovirus cDNA, pMC0 [49]. It resembles GenBank accession DQ294633.1, although the poly(C) tract is absent and there are 13 single-nt differences (A2669C, G3044C, C3371T, A4910C, G4991A, C5156T, G5289A, G5314C, G5315A, A5844C, G6266A, G6990A and A6992G; DQ294633.1 coordinates). The 2B*KO mutation in this genome has been previously described [50]. The TMEV infectious clone is identical to GenBank accession number X56019.1 except for three nt differences, G2241A, A2390G and G4437A [10,51]. All molecular clones were linearized with *Bam*HI, and genomic RNA was *in vitro* transcribed using the T7 RiboMax kit (Promega). Transcripts were purified (RNeasy kit, Qiagen) before being transfected into BHK-21 cells to generate virus stocks.

### Preparation of virus stocks

BHK-21 cells were grown to 60–70% confluency in 10-cm dishes. All Opti-MEM (Gibco) was supplemented with 1:1000 RNaseOUT (Invitrogen). Ninety microlitres of Lipofectamine 2000 (Invitrogen) were added to 1.8 ml Opti-MEM, and 14 µg *in vitro* transcribed genomic RNA was added to another 1.8 ml Opti-MEM. These solutions were mixed and incubated for 20 min whilst cells were washed with PBS. Following the addition of the transfection mixture, cells were incubated for 3.5 h at room temperature with gentle agitation. The transfection medium was removed prior to the addition of 8 ml of 2% FCS DMEM and incubated for 24 h or until full cytopathic effect (CPE) was visible. The plates were then scraped and samples were frozen/thawed thrice, prior to the dead cells and debris being removed by light clarification, and the supernatant was frozen at −70 °C in aliquots. Virus titre was estimated by plaque assay.

### Plaque assays

BSR cells were seeded at 35% confluency in 6-well plates 24 h prior to infection.

Cells were washed once with PBS before the addition of 1 ml sera-free DMEM containing the specified dilution of the virus. The infection was incubated at room temperature with continuous rocking for 1 h before the inoculum was removed, the cells were washed once with PBS and 3 ml of DMEM (2% FCS) containing 1% low melting point agarose (Thermo Fisher Scientific) was added to each well. Cells were incubated for 48 h before being fixed with 4% formaldehyde and stained with toluidine blue. Plaques were counted manually, and their sizes were quantified using ImageJ [52,53].

### Virus infections

BHK-21 or BSR cells were seeded in complete DMEM at ~35% confluence, with the exception of those destined for live-cell imaging assays, which were seeded at 25% confluence. The next day, cells from two spare wells or dishes were trypsinized and counted, with the average of the two wells used to calculate the virus quantity needed for the desired m.o.i. (infectious units estimated by plaque assay). Cells were washed in PBS prior to being infected in FCS-free DMEM, at room temperature with continuous rocking for 1 h to allow adsorption. The infection medium was then removed, and the standard volume of DMEM (2% FCS) was added prior to incubation at 37 °C and 5% CO_2_ for the specified incubation period.

### One-step growth curves

Each specified cell line was infected (m.o.i. 5.0) in confluent 12-well plates. At each timepoint, the medium was removed and clarified by centrifugation at 2500 ***g*** for 5 min before being frozen at −70 °C. Two hundred microlitres of PBS were added to each well, to enable collection of the intracellular virus. Infected cells were frozen/thawed thrice, prior to clarification to remove cellular debris. Viral titre in each fraction was then estimated by plaque assay.

### Luciferase-based cytotoxicity assay

The CytoTox-Glo™ Cytotoxicity Assay (Promega, G9291) was used for these experiments. BHK-21 cells were seeded at ~30% confluency in 10-cm dishes. After 24 h, the cells in a spare plate were counted and frozen in 12 ml DMEM. When thawed and mixed with reagent, this sample would define the luciferase activity, corresponding to 100% cell death. The other plates were infected at an m.o.i. of 5.0. Staurosporine (STS) (1 µM) was added to the apoptosis control plate, and all samples were incubated at 37 °C with 5% CO_2_. At each timepoint, 100 µl of the 12 ml supernatant was taken from each sample and incubated for 15 min at room temperature with 50 µl CytoTox-Glo reagent (Promega), in an opaque 96-well plate. Relative luciferase units (RLUs) were measured with a Glo-Max luciferase reader. A sample of fresh media was used to calculate the RLU for 0% cell death.

### Live-cell imaging

Cells were infected in a 96-well plate (m.o.i. 5.0) in triplicate. Separate wells were used for each reagent applied – green caspase-3/7 (Sartorius, 4440) and NIR Cytotox Dye (Sartorius, 4846) – to avoid spectral overlap. Both reagents were used at a final dilution of 1:1000 in 2% FCS DMEM. When necessary, compounds resuspended in DMSO were added in triplicate to cells infected with each virus. DMSO did not exceed 0.5% (final concentration) in any instance. Cells were imaged every 2 h for a minimum of 24 h, using the Incucyte live-cell imaging and analysis system. Three regions were imaged per well using a 10× objective. The Incucyte 2022B Rev2 GUI software was used to analyse the images. Images with high and low fluorescence and the highest background in each colour were selected to set the background threshold for each plate. Cell confluence was measured by phase microscopy. Caspase-3/7 activity was measured by green colorimetric units (GCUs) per square micromolar, whilst cytotoxicity was measured by NIR count. To allow the normalization of these data to account for changes in cell density, these results were divided by the cell confluence of the same well at 0 hpi.

### Drug treatments

All reagents and compounds were diluted and mixed into separate aliquots of DMEM (2% FCS) immediately prior to use. DMSO-only control wells were included. Compounds, final concentration and source were as follows: STS, 1 µM (Abcam, AB146588); z-VAD-fmk, 50 µM (Promega, G7231); 2-BP, 100 µM (Sigma, 238422); and z-VEID-fmk, 40 µM (Sigma, 218757).

### Flow cytometry

BHK-21 cells were infected (m.o.i. 5.0) in 6-well plates. To increase the percentage of intact cells in the STS-treated control, the STS concentration was reduced to 0.5 µM in this assay. Individual wells were also included as single-stained controls to allow compensation. At each specified timepoint, cells were trypsinized and resuspended in 20% FCS in PBS. The samples were centrifuged at 500 *g* for 5 min before the supernatant was removed. Cells were gently resuspended to reduce cross linking, prior to being fixed in 4% formaldehyde in PBS for 15 min. Cells were then washed twice before being resuspended in fresh PBS. Earlier samples were kept at 4 °C until all samples for the given experimental repeat had been collected and could be immunostained simultaneously. Cells were permeabilized with 90% ice-cold methanol for 10 min and then washed twice prior to immunostaining. Primary antibodies [mouse anti-dsRNA (rJ2, Sigma) (APC-A) and rabbit anti-cleaved caspase-3 (GeneTex) (FITC)] were diluted 1:500 in 0.5% BSA in PBS. Samples were incubated for 1 h at room temperature prior to being washed twice with wash buffer (0.5% BSA, 0.1% TritonX-100 in PBS). Secondary antibodies [goat anti-mouse 633 (Invitrogen A21050) and chicken anti-rabbit 488 (Invitrogen A21441)] were diluted 1:500 and 1:200, respectively, into 0.5% BSA in PBS. Samples were incubated with secondary antibodies for 45 min at room temperature in the dark. Samples were washed twice in wash buffer and then resuspended in 500 µl PBS and analysed immediately using a BD LSR-II flow cytometer at the NIHR Cambridge Biomedical Research Centre. Compensation controls were included for each experiment. Initial analysis was undertaken using BD FACSDiva 8.0.3 software at the time of flow cytometry, prior to a more thorough analysis with FlowJo 3.0. Objects with a higher level of dsRNA or cleaved caspase-3 than that found in the mock (indicated by increased APC-A and FITC, respectively) were considered positive. Gating first compared forward scatter/side scatter to remove any debris and then forward scatter (area)/forward scatter (width) to remove any doublets. To allow analysis of only the infected cells, the single cells of the infected samples were then gated based on the presence of dsRNA, prior to analysis of cleaved caspase-3 levels.

### Transmission electron microscopy

Monolayers of MEF cells were seeded in 3.5-cm dishes with a flat and clear bottom coated with ibidi Polymer. After 24 h, cells from two dishes were counted, and duplicate dishes for each sample were infected at an m.o.i. of 5.0 with WT EMCV, 2B*KO EMCV and TMEV mock-treated or treated with 1 µM STS. At each specified timepoint (7 and 12 hpi), the duplicate samples were washed with PBS before being fixed (fixative: 2% formaldehyde, 2% glutaraldehyde, 50 mM sodium cacodylate pH 7.4 and 2 mM calcium chloride) for 2 h at room temperature and then overnight at 4 °C. After washing 5× with 50 mM sodium cacodylate buffer pH 7.4, samples were osmicated (1% osmium tetroxide, 1.5% potassium ferricyanide and 50 mM sodium cacodylate buffer pH 7.4) for 3 days at 4 °C. After washing 5× in deionized water, samples were treated with 0.1% (w/v) thiocarbohydrazide in water for 20 min at room temperature in the dark. After washing 5× in deionized water, samples were osmicated a second time for 1 h at room temperature (2% osmium tetroxide in water). After washing 5× in deionized water, samples were block stained with uranyl acetate (2% uranyl acetate and 50 mM maleate buffer pH 5.5) for 3 days at 4 °C. Samples were washed 5× with deionized water and then dehydrated in a graded series of ethanol (50%, 70%, 95% and 100%) and 100% dry acetonitrile, three times in each for 5 min. Samples were infiltrated with a dry acetonitrile/Quetol resin mixture [50/50 w/v, without benzyldimethylamine (BDMA)] overnight, followed by 3 days in 100% Quetol (without BDMA). The sample was then infiltrated for 5 days in 100% Quetol resin with BDMA, exchanging the resin each day [12 g Quetol-651, 15.7 g nonenyl succinic anhydride, 5.7 g methyl nadic anhydride and 0.5 g BDMA (TAAB Laboratories Equipment Ltd)]. The resin was cured at 60 °C for 2 days. Thin sections (~80 nm) were prepared using an ultramicrotome (Leica Ultracut E) using a diamond knife and mounted on bare copper grids. Images were collected using an FEI Tecnai transmission electron microscope at an operating voltage of 80 kV, mounted with a Soft Imaging System Megaview III digital camera.

### Immunoblots

All samples to be analysed by immunoblot were frozen in RIPA buffer (ThermoFisher Scientific) containing 1:1000 Benzonase nuclease (Sigma) and protease and phosphatase inhibitors (Halt, ThermoFisher Scientific) (‘complete RIPA’). All samples were boiled in SDS-based protein loading dye (Laemmli buffer) containing 10 mM DTT for 7 min prior to electrophoresis. Following resolution, proteins were transferred to 0.2 µm nitrocellulose membranes by semi-dry electrotransfer in a Transblot turbo transfer system using recommended standard settings (Bio-Rad). Membranes were blocked with 5% (w/v) non-fat milk powder in PBS for a minimum of 3 h with continuous rocking. Primary antibodies were diluted in blocking buffer prior to incubation with the membrane for at least 2 h [mouse anti-FLAG (Sigma, F1084), mouse anti-GAPDH (Abcam, ab125247), rat anti-tubulin (Sigma, MAB1864 YL1/2), rabbit anti-mengo 1D (custom antibody against peptide NGHKRFDNTGDLGI, GenScript) and rabbit anti-2B* (custom antibody, GenScript [4]]. Membranes were washed three times with TBS-Tween 20 (0.1%) before being incubated with the relevant secondary antibody for 1 h with continuous rocking ([goat anti-mouse 680RD, goat anti-rat 680RD and goat anti-rabbit 800CW (LI-COR)]. Membranes were again washed three times with TBS-Tween 20 (0.1%) before being imaged with the Odyssey CLx imaging system (LI-COR).

## Results

### Three conserved motifs within the C-terminal domain of 2B* contribute to a large plaque phenotype

Various 2B*KO viruses have been previously described [4,7], all of which produce small plaques during infection of BHK-21 cells. One of these so-called knockouts actually encodes a truncation, as it possesses two premature termination codons in the 2B* ORF (without changing the 2B aa sequence), resulting in the translation of a 2B*-derived peptide 63 residues in length [4]. Thus, the expression of just the N-terminal half of 2B* is insufficient for WT plaque sizes. Following aa alignment of 2B* from multiple EMCV sequences (Fig. 2a), we selected three regions of increased conservation within the C-terminal half and engineered three mutant viruses, each of which had one of these motifs disrupted in 2B*. All mutations were chosen to not change the aa sequence encoded in the overlapping 2B frame. The three viruses were named as follows: TriKtoE (possessing K61E, K69E and K71E mutations, numbers refer to the 2B* aa sequence), QCADtoPRSH (Q86P, C88R, A89S and D91H) and TriDtoH (D91H, D94H and D99H). A plaque assay performed with the rescued viruses revealed that all three produced small plaques, similar in size to those from a previously characterized 2B*KO EMCV, which encodes a 2B* peptide of only 29 aa [7] (Fig. 2b). BSR cells, a clonal derivative of BHK-21 cells, were used in this assay due to their ability to support clearly defined plaques [54]. We have shown previously that this 2B*KO EMCV genome replicates to WT levels and the mutation does not affect ribosomal frameshifting [7]. This indicates that the C-terminal domain of 2B* contributes to its tertiary structure and/or function and is essential for the WT EMCV phenotype.

### 2B* is required for efficient lytic virus release

**Fig. 3.**
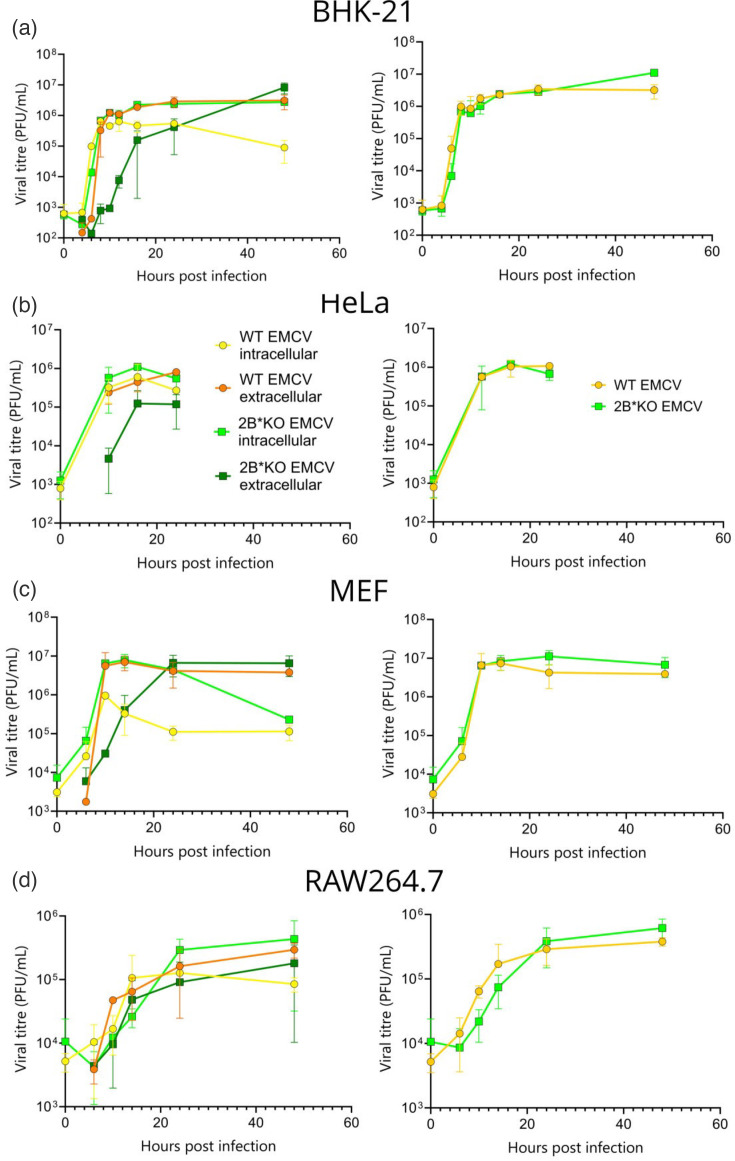
2B*KO EMCV exhibits impaired extracellular virus titres in a variety of cell lines. Confluent monolayers of BHK-21 (**a**), HeLa (**b**), MEF (**c**) and RAW264.7 (**d**) cells were infected with either WT EMCV or 2B*KO EMCV at an m.o.i. of 5.0 as specified. At the indicated timepoint, the medium was removed and frozen separately from the cells. The intracellular and extracellular viral titre in each sample was measured by plaque assay and normalized by the number of cells infected at 0 hpi (viral p.f.u. in each fraction per 10 000 cells) (left-hand panels). Total virus titres were calculated from the measured intracellular and extracellular titres (right-hand panels). Data represent the mean±sem of two independent biological repeats.

Previous studies have reported that the overall titres of 2B*KO EMCV are equivalent to WT, despite the small plaque phenotype [4]. To further investigate this, we infected a range of cell lines with WT EMCV and 2B*KO EMCV and conducted one-step growth curves. Samples were taken at multiple early timepoints, as WT EMCV infection is known to induce significant CPEby 8 hpi in both BHK-21 and HeLa cells [31,32]. However, we separated the extracellular from the intracellular virus at each timepoint, in contrast to previously published work. Using this approach, we saw that 2B*KO EMCV displayed noticeably lower extracellular titres in three of the four cell lines tested until 16 hpi (Fig. 3a–d, left-hand panels), despite the total virus titre being approximately equal between WT EMCV and 2B*KO EMCV (Fig. 3a–d, right-hand panels). At later timepoints, WT EMCV displayed lower intracellular virus titres compared to 2B*KO EMCV. This led us to hypothesize that 2B* contributes to efficient virus egress.

**Fig. 4.**
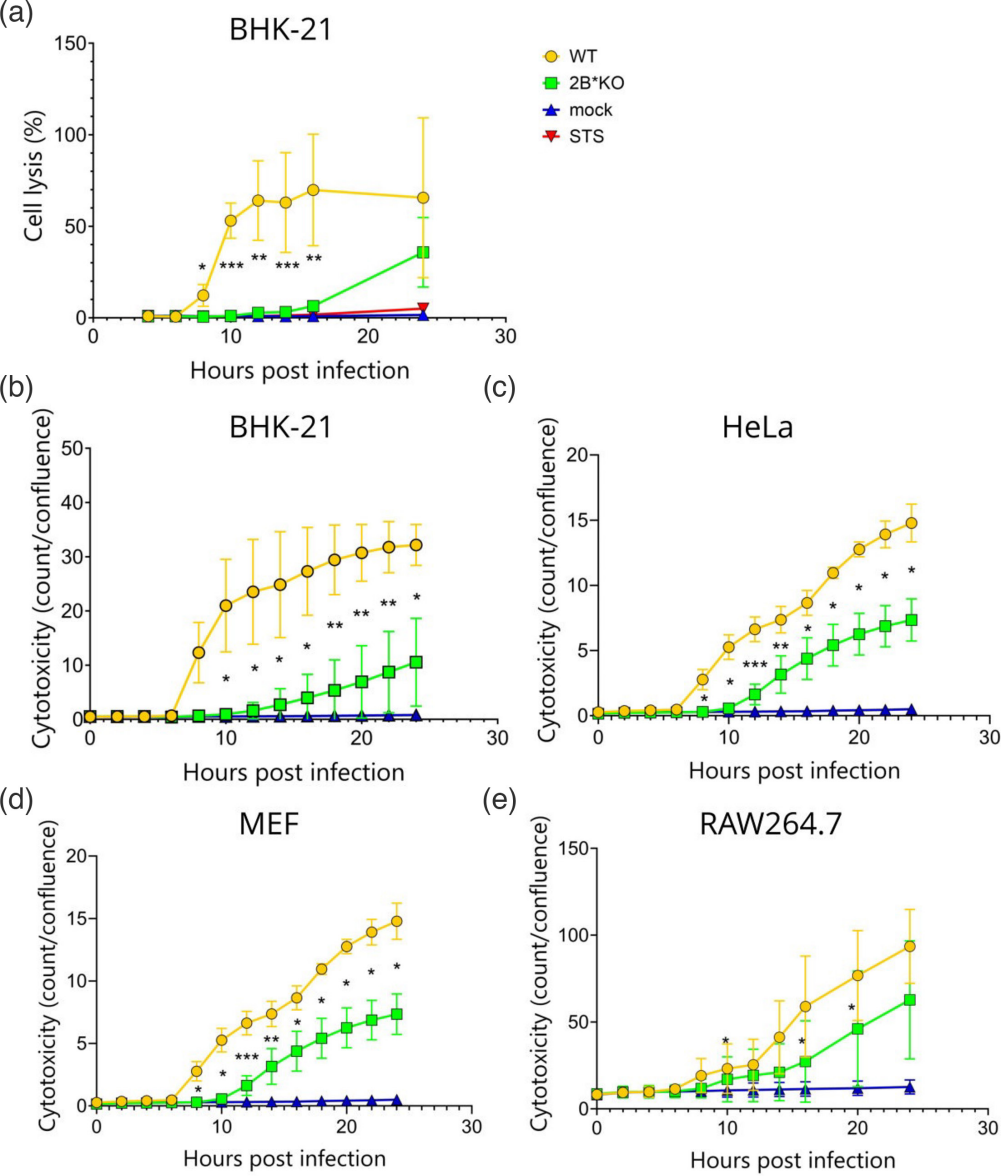
2B*KO EMCV exhibits impaired cell lysis relative to WT EMCV. Confluent monolayers of BHK-21 (a, b), HeLa (c), MEF (d) and RAW264.7 (e) cells were infected with either WT EMCV or 2B*KO EMCV at an m.o.i. of 5.0 or treated with STS (0.5 µM) (a only) as specified. The cytotoxicity in each sample was measured over time either using a luciferase-based assay (a) or an NIR object count (count) by live-cell imaging in triplicate wells using the Incucyte (Sartorius) live-cell microscope and analysis software and adjusted for cell confluence (b–e). Data shown are the mean±sd of four (a) or three (b–e) biological repeats, each using triplicate wells. Statistical analysis [ratio-paired t-test (a) and Student’s t-test (b–e)]: *P≤0.05, **P≤0.01, ***P≤0.001. Significance values are shown relative to WT EMCV.

Despite relatively minor contributions from non-lytic virus release [13,55,60], picornaviruses primarily exit the infected cell by inducing lysis. We therefore compared the cytotoxicity of WT and 2B*KO EMCV infection over 24 h, as this would be expected to correlate with the level of lytic virus release. A luciferase-based assay, which measured the activation of a luciferase substrate following cleavage by released cellular proteases, revealed that significant cell lysis commenced at 8 hpi during WT EMCV infection of BHK-21 cells (Fig. 4a). This timing correlates with the peak of 2B* production in BHK-21 cells [6]. However, lysis was delayed until 16 hpi in 2B*KO EMCV-infected BHK-21 cells (Fig. 4a). The inclusion of an uninfected, STS-treated sample confirmed that the assay could not differentiate cells undergoing apoptotic cell death from living cells. The switch-like temporal regulation of lysis during WT EMCV infection, soon after 2B* is expressed and greatly delayed lysis during 2B*KO EMCV infection, indicated that the temporal regulation of frameshifting also acts to temporally regulate viral lytic release.

These results were reproduced in a range of other cell lines, using a live-cell imaging assay, which measured the ability of a reagent to enter the cell following the loss of plasma membrane integrity, inducing fluorescence. This alternative assay was chosen due to its high-throughput nature, low cost and ease of use. These experiments indicated that 2B*KO EMCV infection was accompanied by significantly impaired cell lysis rates compared to WT EMCV infection, regardless of the cell type (Fig. 4b–e). This led us to hypothesize that 2B* plays a direct role in enabling EMCV viral spread by lytic release, explaining the small plaque phenotype reported in all engineered 2B*KO viruses to date [4,7], as well as the small plaques observed in our 2B* mutant viruses (Fig. 2b). The delayed cell lysis in 2B*KO EMCV infections correlates with the reduction in released virus at earlier timepoints. However, at this stage, we could not eliminate an anti-apoptotic function of 2B* (which has been previously attributed to 2A), as the viral entrapment caused by apoptosis would reduce virus release and would not be detected by the cytotoxicity assay used, as shown by the lack of detectable lysis in the STS-treated sample (Fig. 4a). The increase in both virus release and plaque size was clearly caused by an increase in lytic cell death and not primarily due to a role in promoting non-lytic release.

### 2B*KO EMCV-infected cells do not undergo apoptosis

To further investigate whether 2B*KO EMCV-infected cells were undergoing apoptosis, we performed transmission electron microscopy (TEM) of infected MEF cells to assess the effects of infection on cellular ultrastructure. MEF cells were chosen due to their amenability to the method, whereas BHK-21 cells proved difficult to image. Knockout of 2A has also been linked to apoptosis induction, but, since the removal of 2A would also have inadvertently inhibited PRF and hence prevented 2B* expression, it is possible that apoptosis induction is a consequence of loss of 2B* rather than loss of 2A [30,32, 33]. The viral protease 3C^pro^ is also a well-established anti-apoptotic viral protein [21]; it is therefore clear that WT EMCV has a multitude of mechanisms in place to avoid this route of cell death. Signs of early apoptosis include cell rounding, fragmentation of the cell into membrane-bound vesicles (apoptotic bodies), cytosolic condensation, nuclear fragmentation and membrane blebbing [61]. As TMEV is one of the closest relatives of EMCV, yet does not possess 2B*, it was included for additional comparison. Cells were infected with WT EMCV, 2B*KO EMCV or TMEV at an m.o.i. of 5 and fixed at 7 hpi (WT EMCV only) or 12 hpi, before being prepared for TEM. WT EMCV-infected cells were harvested at the earlier timepoint to avoid loss of the sample, as almost total lysis was observed by 12 hpi. STS-treated cells were included to assess the induction of apoptosis.

**Fig. 5.**
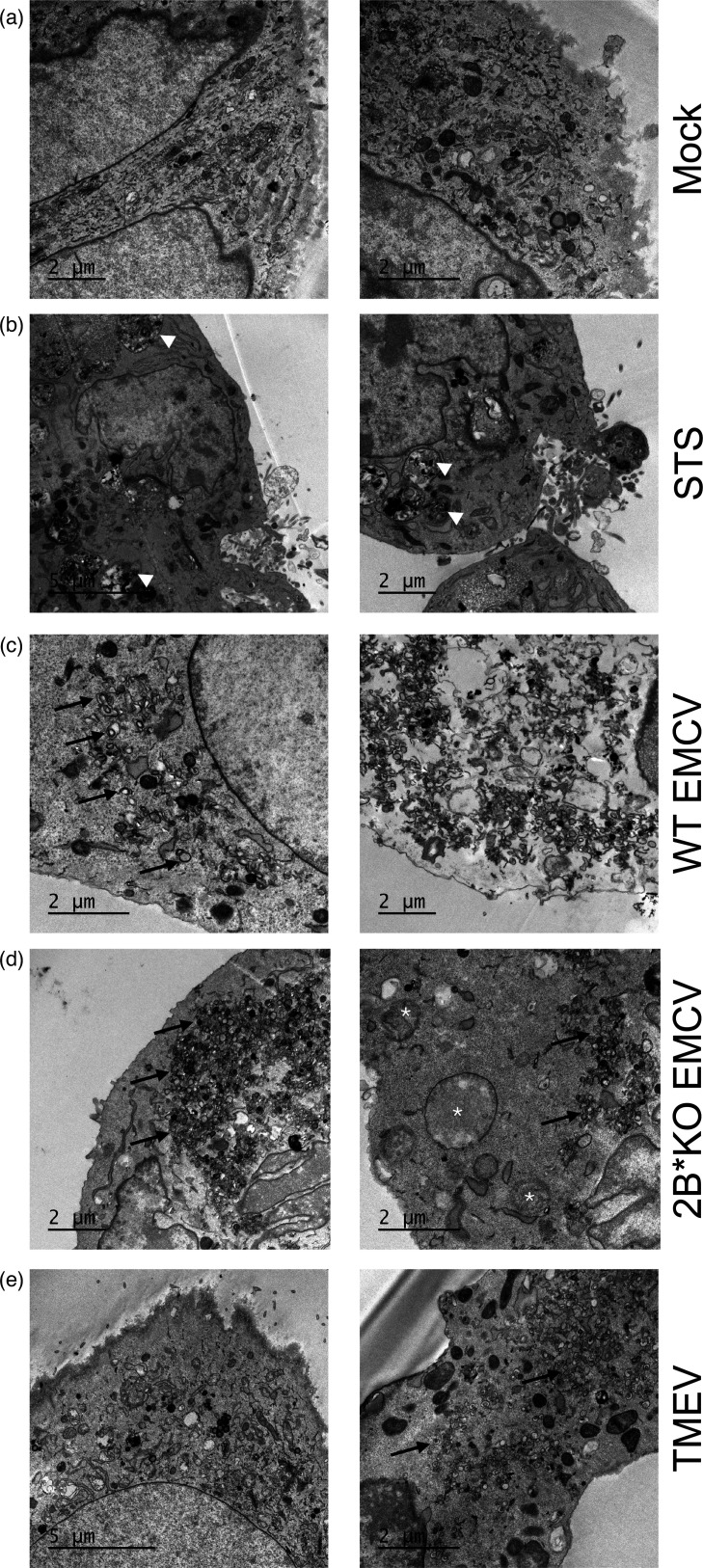
2B*KO EMCV-infected cells are morphologically distinct from both WT EMCV-infected and TMEV-infected MEF cells. MEF cells were either mock infected (**a**), treated with 1 µM STS (**b**) or infected at an m.o.i. of 5.0 with WT EMCV (**c**), 2B*KO EMCV (**d**) or TMEV (**e**). Electron micrographs show vacuoles (white arrowheads) in STS-treated cells, replication organelles (black arrows) in all infected samples and enlarged mitochondria with unusual round appearance (asterisks) in 2B*KO EMCV-infected cells. Electron lucent cytosol, indicating a loss of membrane permeability, is visible in WT EMCV-infected cells (right-hand panel). The WT EMCV-infected cells were harvested at 7 hpi; all other samples were harvested at 12 hpi. Images are two representative images of one sample, harvested at the same timepoint.

Ultrastructural analysis by TEM revealed that STS-treated cells displayed condensed and dense cytosol, with the presence of multiple vacuoles (Fig. 5b, white arrows), which were not a feature upon either WT (Fig. 5c) or 2B*KO EMCV (Fig. 5d) infection but are known features of apoptosis [61]. Viral replication organelles were clearly present in WT EMCV-, 2B*KO EMCV- and TMEV-infected cells (Fig. 5c–e, black arrows), confirming cell infection.

Unlike the WT EMCV-infected cells that were undergoing cellular permeabilization and release of the cytosol (Fig. 5c), 2B*KO EMCV-infected cells did not show any morphological signs associated with the late stages of apoptosis at this time. As expected based on the cytotoxicity assay, the plasma membrane of the 2B*KO EMCV-infected cells was still intact, as indicated by the retention of the cytosol. Mitochondria were enlarged and more spherical in 2B*KO EMCV-infected cells (Fig. 5d, asterisks) compared to other samples. Interestingly, the TMEV-infected cells (Fig. 5e) looked markedly different from the 2B*KO EMCV-infected cells, with TMEV-infected cells displaying mitochondria with normal morphology. Whilst the changes to mitochondrial morphology and the lack of membrane permeabilization in the 2B*KO EMCV-infected cells may indicate that 2B* has an anti-apoptotic role, the lack of other morphological signs makes this unlikely. We hypothesized that the 2B*KO EMCV-infected cells were initiating early intrinsic apoptosis before being redirected to another cell death pathway, rather than continuing to the late stages of apoptosis.

**Fig. 6.**
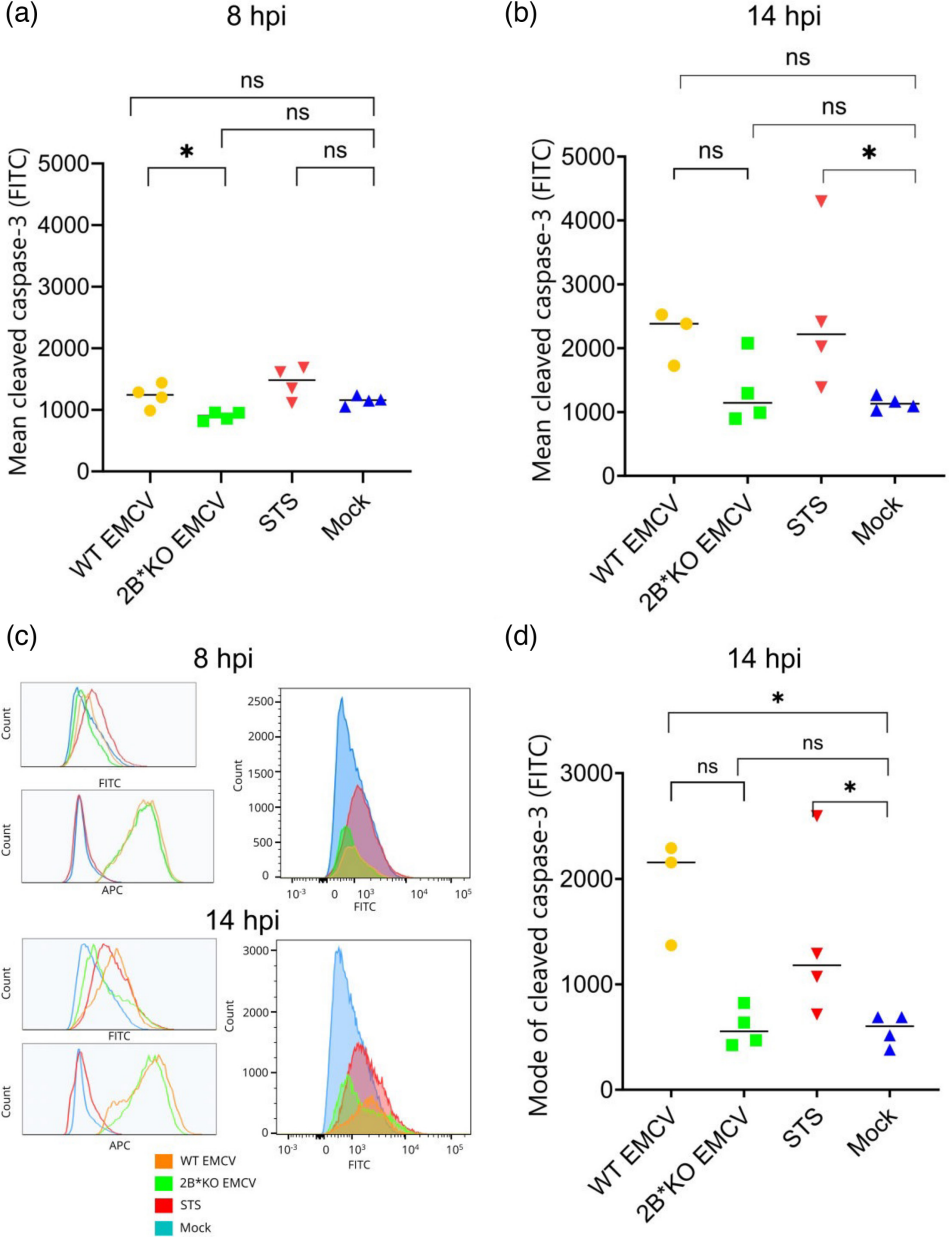
WT EMCV induces more cleavage of caspase-3 than 2B*KO EMCV at timepoints, which showed differences in cell lysis. BHK-21 cells were infected with WT EMCV or 2B*KO EMCV at an m.o.i. of 5.0, mock infected or treated with 0.5 µM STS. Cells were fixed at 8 hpi (a) or 14 hpi (b), permeabilized and stained with antibodies recognizing dsRNA (APC) or cleaved-caspase-3 (FITC). Samples were analysed using an LSR-II flow cytometer. Data shown are the indicated average (mean or mode) of cleaved caspase-3 in a minimum of three independent biological repeats. Statistical analysis (a, b, d) (Mann–Whitney *U* test) was used to compare the mean cleaved caspase-3 in the infected single cells, or the single cells in the mock-infected and STS-treated control samples. ns, not significant, *P≤0.05. (c) Histograms show the relative amount of dsRNA and cleaved caspase-3 in each cell in each sample of one biological repeat; data are representative of the three biological replicates. (d) The mode level of cleaved caspase-3 detected in each cell, in each sample, fixed at 14 hpi. Horizontal lines indicate the mean of the modes for each sample. Note that the effect from STS (the positive control) only becomes statistically significant at 14 hpi.

To further clarify whether apoptosis was initiated in 2B*KO EMCV-infected cells, we investigated caspase-3 activation, commonly regarded as being the hallmark of apoptosis induction. Caspase-3 is known to be cleaved during EMCV infection [62]. We envisioned two scenarios: if 2B* protects against apoptosis, then 2B*KO EMCV-infected cells would exhibit a marked increase in cleaved caspase-3 earlier than would be seen in WT EMCV-infected cells. However, if 2B* is acting to induce caspase-3-dependent (GSDME-mediated) pyroptosis (which WT EMCV is known to cause [34]), then 2B*KO EMCV-infected cells would have less cleaved caspase-3 than WT EMCV-infected cells, at timepoints where lysis is reduced in 2B*KO EMCV infection. We measured cleaved (activated) caspase-3 by flow cytometry and found that at the earlier timepoint of 8 hpi, 2B*KO EMCV infection was in fact accompanied by lower cleaved caspase-3 levels than WT EMCV (Fig. 6a). This trend was noticeable but not statistically significant at the later timepoint of 14 hpi (Fig. 6b–d). The reduced height of the histogram for the WT EMCV-infected cells (Fig. 6c) indicates that there were fewer cells remaining in this sample at this time, which was expected due to the majority of WT EMCV-infected cells having undergone cell lysis by 14 hpi.

From these experiments, we can conclude that, in direct contrast to inducing apoptosis (which would correlate with reduced lysis [31]), the loss of 2B* results in delayed caspase-3 activation. Hence, 2B* is unlikely to be an anti-apoptotic protein, as caspase-3 activation, a hallmark of apoptosis, is not enhanced in 2B*KO EMCV-infected cells. Despite greatly delayed cell lysis, 2B*KO EMCV infection clearly did not result in late-stage apoptosis, and we therefore concluded that 2B* is involved in cell lysis induction.

### WT EMCV-induced lysis requires both 2B* and caspases for maximum efficacy

**Fig. 7.**
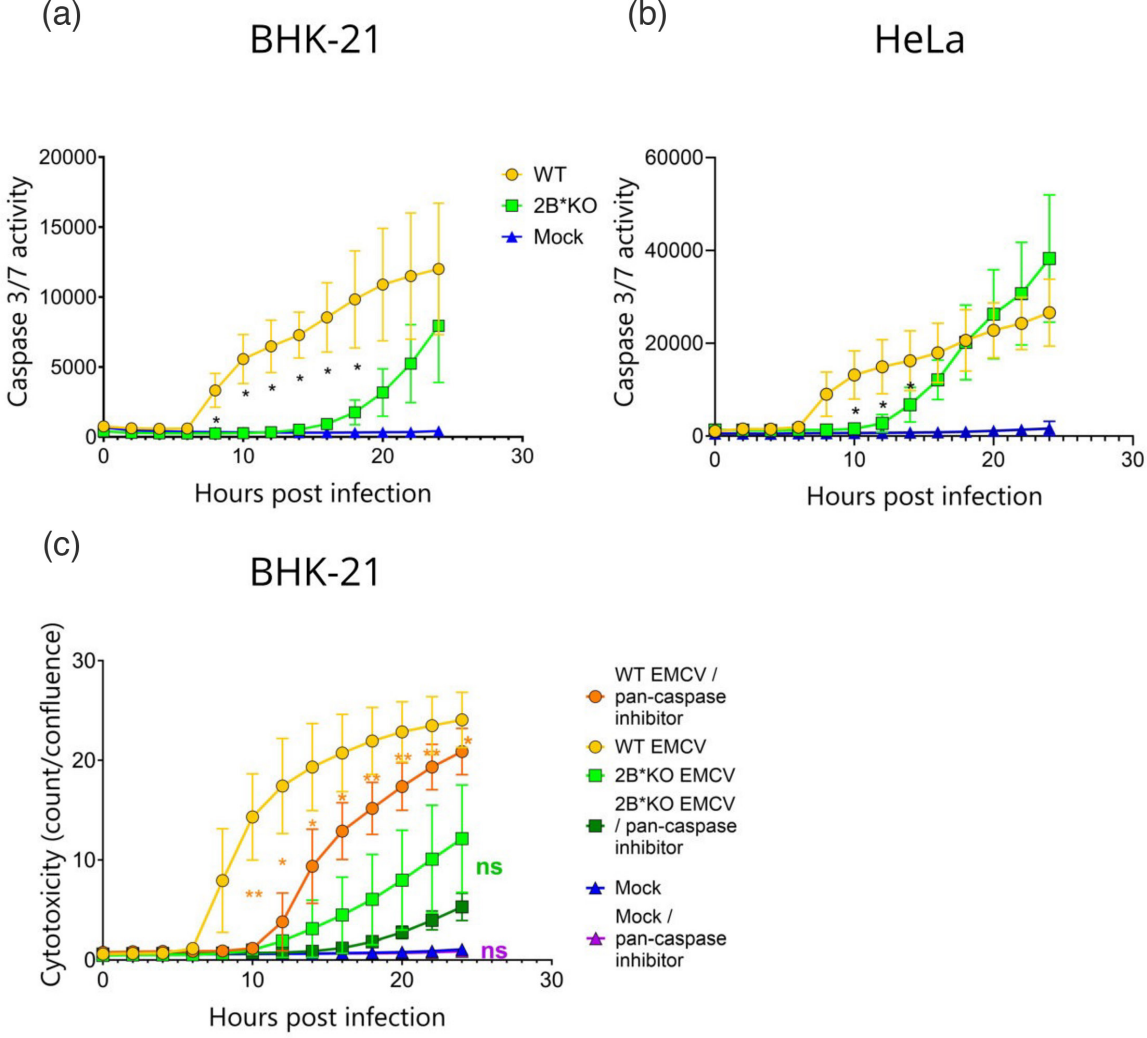
Efficient WT EMCV-induced cytotoxicity requires both caspase-3 and 2B*. Confluent monolayers of BHK-21 (a) or HeLa (b) cells were infected with either WT EMCV or 2B*KO EMCV at an m.o.i. of 5.0. The caspase-3/7 activity was measured in GCU per square micromolar by live-cell imaging in triplicate wells using the Incucyte (Sartorius) live-cell microscope and analysis software and adjusted for cell confluence. (c) Confluent monolayers of BHK-21 cells were infected with either WT EMCV or 2B*KO EMCV at an m.o.i. of 5.0 immediately prior to treatment with the pan-caspase inhibitor, zVAD-fmk. The cytotoxicity was measured in NIR object count (count) by live-cell imaging in triplicate wells using the Incucyte (Sartorius) live-cell microscope and analysis software and adjusted for cell confluence, also measured by the Incucyte. Data shown are the mean±sd of three biological repeats, each using triplicate wells. Statistical analysis (Student’s t-test): ns, not significant *P≤0.05, **P≤0.01. All statistical analyses compare the inhibitor-treated samples to the untreated sample infected with the same virus: orange asterisks represent the statistical comparison of cytotoxicity induced by infection with WT EMCV (treated with a pan-caspase inhibitor) compared to WT EMCV (untreated). Green text (ns) indicates 2B*KO EMCV infection (treated with a pan-caspase inhibitor) compared to 2B*KO EMCV (untreated). Purple text (ns) indicates mock-infected cells (treated with a pan-caspase inhibitor), compared to mock-infected cells (untreated).

We next quantified the activation of caspase-3 and caspase-7 during 24 h of infection with either WT or 2B*KO EMCV. In this system, cleaved caspase-3/7 binds to a conjugated, initially inert dye, cleaving the dye, which is then free to stain the nuclear DNA of pro-apoptotic cells. This live-cell imaging approach confirmed that WT EMCV infection caused significantly higher levels of caspase-3 and/or caspase-7 cleavage than 2B*KO EMCV, until 18 hpi in BHK-21 cells and 14 hpi in HeLa cells (Fig. 7a, b). Inhibition of these caspases with a pan-caspase inhibitor reduced WT EMCV-induced cytotoxicity significantly, although interestingly not to the level seen for 2B*KO EMCV infection without the drug (Fig. 7c). The lowest cytotoxicity level was seen for 2B*KO EMCV infection in the presence of the pan-caspase inhibitor. These data confirm that WT EMCV utilizes a caspase-dependent mechanism of cell death, as suggested by previous work [34].

**Fig. 8.**
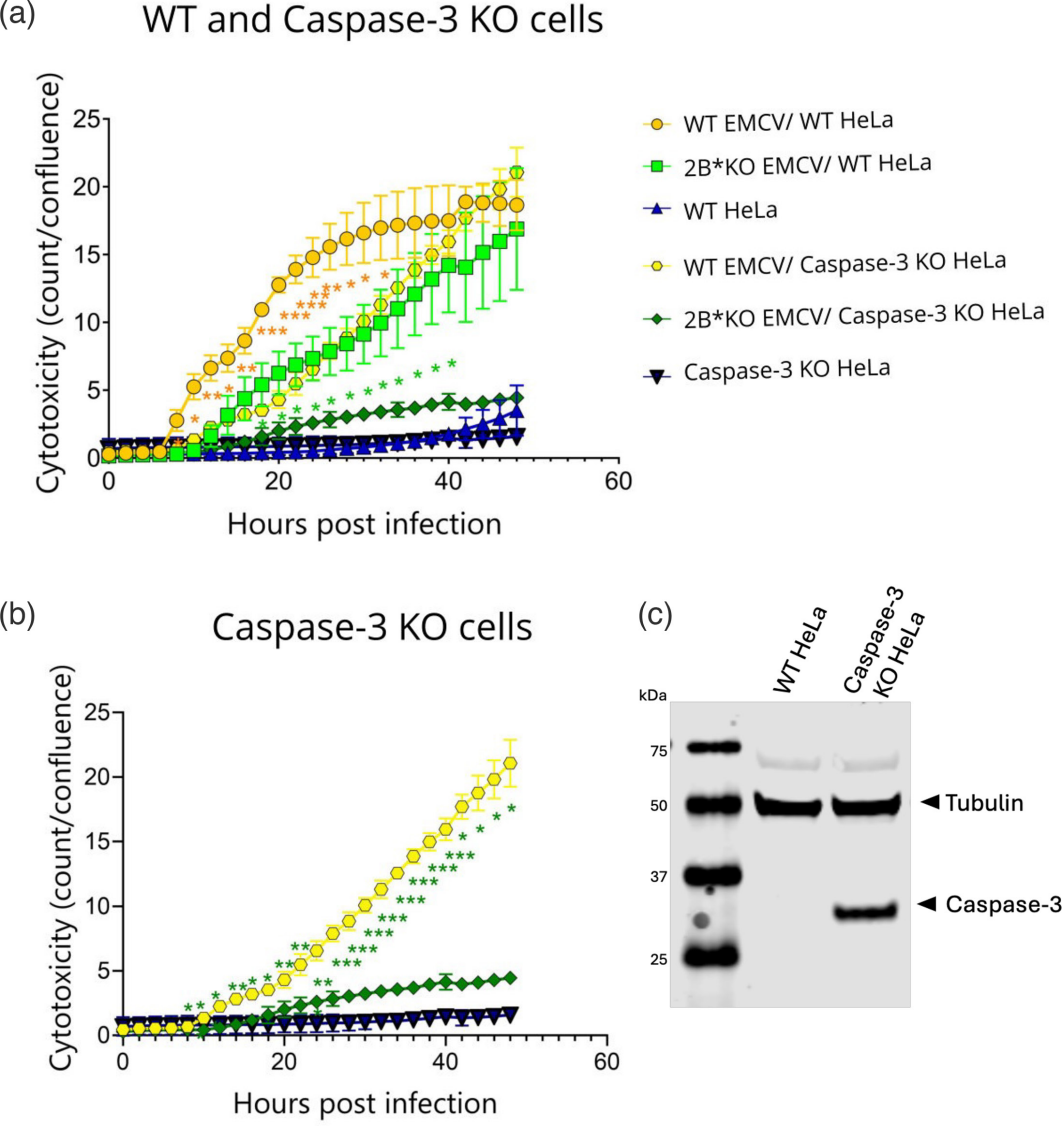
Knockout of caspase-3 reduces both WT and 2B*KO EMCV-induced cell lysis. Monolayers of WT or caspase-3 KO HeLa cells were infected with either WT EMCV or 2B*KO EMCV at an m.o.i. of 5.0. Live-cell imaging was used to measure the cell death in each sample by NIR object count (count). NIR was analysed using the Incucyte live-cell imaging suite (Sartorius) and normalized to the cell confluence determined by the same software. The same data in (**a**) are represented in (**b**), following the removal of the WT HeLa cell samples for clarity. Data shown are the mean±sd of three biological repeats, each using triplicate wells. Statistical analysis (unpaired Welch t-test): *P≤0.05, **P≤0.01, ***P≤0.001. Statistical analyses (**a**) compare the cytotoxicity induced by the same virus in each cell line. Orange asterisks represent the statistical comparison of cytotoxicity induced by infection with WT EMCV, in WT HeLa cells compared to caspase-3 KO HeLa cells. Green asterisks indicate infection with 2B*KO EMCV, in WT HeLa cells compared to caspase-3 KO HeLa cells. (**b**) Statistical analyses compare the cytotoxicity induced by WT EMCV to 2B*KO EMCV in caspase-3 KO HeLa cells only (green asterisks). (**c**) The absence of caspase-3 in the KO HeLa cells was confirmed by immunoblot.

This model was supported by subsequent experiments, utilizing caspase-3 KO HeLa cells [48]. The loss of caspase-3 in these cells reduced the efficiency with which WT EMCV induced lysis, to levels approximately equivalent to those of WT (parental) HeLa cells infected with 2B*KO EMCV (Fig. 8a, see also Fig. 8b for clarity). However, neither of these lysis rates were as low as that produced when the caspase-3 KO HeLa cells were infected with 2B*KO EMCV (i.e. dual loss of both caspase-3 and 2B*), indicating that at least one alternative, caspase-3-independent pathway, can be utilized. Strikingly, this indicates that WT EMCV infection in BHK-21 cells can activate at least two distinct pathways, leading to cell lysis and death, both of which require 2B* for maximum efficiency: one caspase-3 dependent and the other caspase-3 independent. The inefficient cell lysis observed during 2B*KO EMCV infection correlates with the poor extracellular virus titres produced by multiple cell types (Fig. 3).

The combined presence of 2B* and caspase-3 is clearly necessary for maximum levels of cell lysis. However, in the absence of caspase-3, WT EMCV-induced cell lysis was approximately equivalent to that of 2B*KO EMCV in the presence of caspase-3, in the HeLa cells. This created a discrepancy between the two cell lines since in BHK-21 cells, there were clearly four levels of cytotoxicity across the virus±pan-caspase inhibitor permutations (Fig. 7c). This may be due to different expression levels of the relevant host factors or efficacy of the drug, which was difficult to assess in hamster cells. However, regardless of the cell type, it appears that EMCV can consistently induce cell lysis via two distinct pathways: one caspase-3 dependent and the other caspase-3 independent. 2B* increases the efficiency of both, indicating that it is a key driver in cell lysis during EMCV infection.

### 2B* is required for efficient GSDME-mediated pyroptosis during EMCV infection

WT EMCV is known to induce cleavage of overexpressed GSDME via caspase-3 activation [34]. EV71 and FMDV have also recently been shown to utilize GSDME-mediated pyroptosis to induce cell lysis [44,45], indicating that the activation of this pathway may be a conserved feature of picornavirus infections, even if the activation mechanisms differ. We therefore hypothesized that the caspase-3-dependent cell death pathway activated by WT EMCV in our assays was GSDME-mediated pyroptosis.

**Fig. 9.**
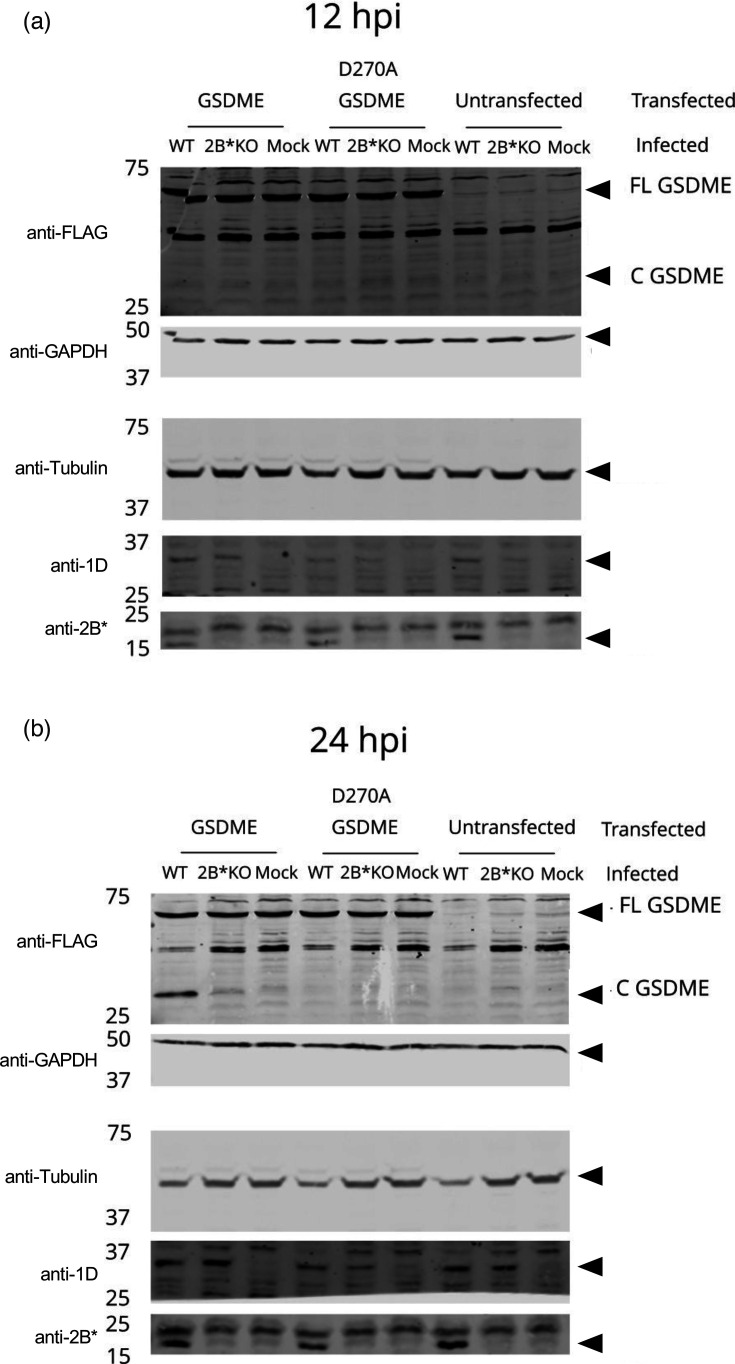
2B* is required for efficient GSDME cleavage during EMCV infection. HEK293T cells were transiently transfected with pcDNA3.1-myc-GSDME-FLAG, or an uncleavable mutant GSDME (D270A), 24 h prior to infection with either WT EMCV or 2B*KO EMCV at an m.o.i. of 5.0. At the specified timepoints, 12 hpi (a) and 24 hpi (b), samples were frozen in a complete RIPA buffer. Proteins were resolved on two separate SDS-PAGE gels, followed by immunoblotting with the specified antibodies. Immunoblots shown are representative of two independent biological repeats. FL GSDME, full-length GSDME; C GSDME, C-terminal fragment of GSDME.

The role of 2B* in promoting GSDME-mediated pyroptosis has not been investigated, and it clearly could not be required for this process for any other picornavirus, as only EMCV encodes 2B*. To investigate whether 2B* was required for efficient execution of this pathway during EMCV infection, we infected HEK293T cells, which had been transiently transfected with a tagged GSDME construct. HEK293T cells were chosen due to the high transfection efficiency that could be achieved. An uncleavable mutant GSDME (D270A) was included as an additional control, to confirm specificity [63]. By 24 hpi, GSDME was cleaved much more efficiently in WT EMCV infection compared to 2B*KO EMCV infection (Fig. 9), indicating that 2B* is important for efficient utilization of this pathway in HEK293T cells.

We tested all the relevant cell lines for GSDME expression (Fig. S1, available in the online Supplementary Material) and confirmed the expression in all except BHK-21 and their clonal derivative, BSR cells. This result was however inconclusive since the hamster GSDME mRNA NCBI accession, XM_005087183.4, is only a predicted mRNA, and the epitope recognized by the commercial antibody was predicted to possess only 72% identity to the hamster homologue. Therefore, at this stage, we assume that the hamster cell lines in question do express GSDME, given that the small plaque phenotype was originally observed in these cells and they exhibited similar cytotoxicity profiles to HeLa cells, where we confirmed the expression.

**Fig. 10.**
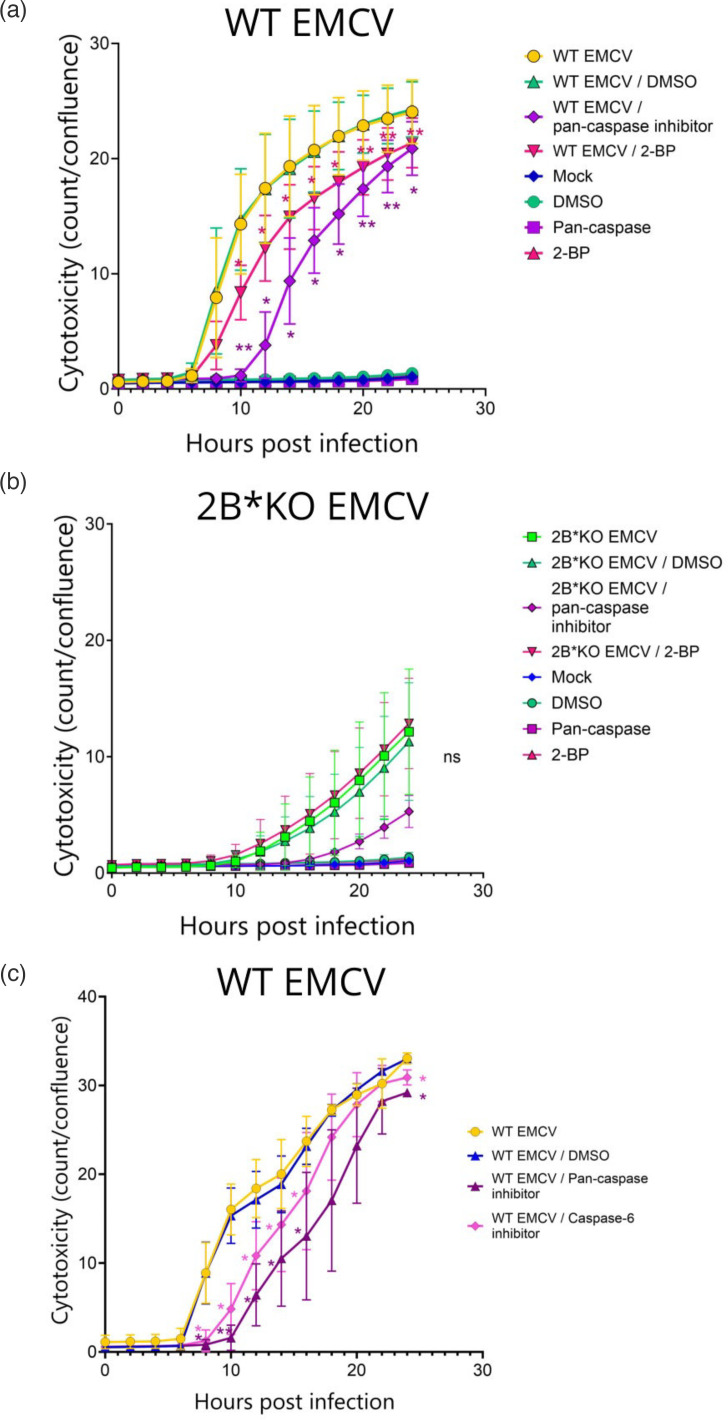
Inhibition of either palmitoylation or caspase-6 activation reduces WT EMCV-induced cell lysis. Monolayers of BHK-21 cells were infected with WT EMCV (**a**) or 2B*KO EMCV (**b**) at an m.o.i. of 5.0 prior to the addition of either the palmitoylation inhibitor 2-BP or the pan-caspase inhibitor zVAD-fmk. (**c**) Monolayers of BHK-21 cells were infected with WT EMCV at an m.o.i. of 5.0 prior to the addition of the specified inhibitor. Live-cell imaging was used to measure the cell death in each sample by NIR object count (count). NIR was analysed using the Incucyte live-cell imaging suite (Sartorius) and normalized to the cell confluence determined by the same software. Data shown are the mean±sd of three biological repeats, each using triplicate wells. Statistical analysis (Student’s t-test): ns, not significant, *P≤0.05, **P≤0.01. All statistical analyses compare the inhibitor-treated samples to the untreated sample.

GSDME-mediated pyroptosis is reliant upon palmitoylation of the C-terminal fragment. Therefore, we next investigated the effect of the palmitoylation inhibitor 2-BP, known to inhibit GSDME function [64], upon EMCV-induced cytotoxicity in BHK-21 cells. It must be noted that, due to its nonspecific nature, this drug would have significant off-target effects upon the host cell membranes, including the mitochondria, which were affected during 2B*KO EMCV infection (Fig. 5d) [65]. We found that the inhibition of palmitoylation by 2-BP significantly impaired cell lysis induced by WT EMCV, but not that induced by 2B*KO EMCV in BHK-21 cells (Fig. 10a, b), providing supporting evidence that WT EMCV induces GSDME-mediated pyroptosis and 2B* is required for efficient utilization of this pathway.

GSDME cleavage would also be induced by caspase-6 activity, as this acts upstream of caspase-8 in the intrinsic apoptosis pathway; caspase-6 has also been shown to inefficiently cleave GSDME directly [66,67]. Therefore, to provide further supporting evidence that this pathway was being utilized by WT EMCV, we investigated whether targeting the upstream-acting caspase-6, by treatment with the caspase-6 inhibitor (Z-VEID-fmk), affected cytotoxicity in WT EMCV-infected BHK-21 cells. We observed a consistent reduction in cell lysis in the infected treated cells, indicating that caspase-6 is indeed important for efficient cell death (Fig. 10c). However, it remains possible that caspase-6 is also or instead activating an alternative, as-yet-unidentified caspase-3-independent pathway, as it does not specifically target caspase-3.

## Discussion

Here, we show that 2B* increases the efficiency of lytic virus release during EMCV infection (Figs3  4) by inducing caspase-3 activation (Figs6  7) and caspase-3-mediated cell lysis (GSDME-mediated pyroptosis, Fig. 9). In addition, 2B* promotes an alternative, caspase-3-independent mechanism of cell lysis, which remains unidentified (Fig. 8). Whilst 2B*KO EMCV can induce cell lysis during infection, it does so far less efficiently than WT EMCV, limiting cell-to-cell spread and resulting in the characteristic small plaque phenotype reported previously [4,6, 7] and recapitulated here by multiple new mutants (Fig. 1). We conclude that 2B* functions to enable the rapid cell lysis characteristic of EMCV infection. This further confirms that 2B* is a multi-functional accessory protein [7] and not a mere by-product of a functionally important ribosome frameshift event. This work contributes to the understanding of EMCV pathogenesis by identifying 2B* as a source of increased efficiency in EMCV-induced cell lysis.

In accordance with previous reports, we found that WT EMCV primarily utilizes a caspase-3-reliant lytic pathway to mediate virus release (GSDME-mediated pyroptosis) [34] (Fig. 9b). By comparison, GSDME cleavage during 2B*KO EMCV infection was drastically reduced, consistent with the reduced cytotoxicity observed during 2B*KO EMCV infection at this timepoint (Fig. 4). The decreased efficiency of GSDME cleavage during 2B*KO EMCV infection may therefore be the primary cause of the impaired viral lytic release phenotype, corresponding to the formation of small plaques. It appears that 2B* is essential for the efficient activation and execution of this pathway.

However, we also observed that WT EMCV utilizes an alternative, caspase-3-independent pathway, when GSDME-mediated pyroptosis is inaccessible through either caspase-3 KO (Fig. 8a) or inhibition (Fig. 7c). Similar to the previous scenario, 2B* is required for efficient activation of this pathway, as 2B*KO EMCV was unable to induce substantial amounts of lysis even by 48 hpi in the caspase-3 KO HeLa cells (Fig. 8b). These data suggest that (i) 2B*KO EMCV is almost entirely reliant on caspase-3 to induce lysis in HeLa cells, despite not inducing high levels of GSDME cleavage, and (ii) 2B* also enables WT EMCV to efficiently utilize an alternative, caspase-3-independent pathway.

One obvious question is whether the caspase-3-dependent pathway utilized by 2B*KO EMCV is delayed GSDME-mediated pyroptosis (which may have been undetectable using our current approaches) or another pathway. In both WT and 2B*KO EMCV infections, the activation of caspase-3 may be caused by the endoplasmic reticulum stress produced by 2B [68] and/or the upregulation of NOXA [21], both of which have previously been associated with EMCV-induced cell lysis [69]. However, the levels of cleaved caspase-3 induced by 2B*KO EMCV (Fig. 6) may never reach the threshold required for GSDME-mediated pyroptosis.

The low levels of cleaved caspase-3 induced by 2B*KO EMCV infection also make it clear that 2B* is not simply an ‘anti-apoptotic’ protein, preventing WT EMCV-infected cells from completing apoptosis. In comparison, L, 2A and 3C^pro^ all possess distinct anti-apoptotic functions, although the phenotypes differ between cell lines [21,30, 31, 33]. On the contrary, 2B* promotes caspase-3 activation, as WT EMCV induced rapid caspase-3 and/or caspase-7 cleavage at 8–12 hpi whereas 2B*KO EMCV-induced caspase activation was delayed in both cell lines tested (Fig. 7a, b). We have no evidence that 2B* mediates caspase-3 activation via a direct interaction; 2B* may instead activate an upstream regulatory protein, initiating multiple cell death pathways (see further discussion below). The mechanism by which caspase-3 balances apoptosis against GSDME-mediated pyroptosis induction is currently unknown. In the case of EMCV infection, it is possible that 2A is responsible for this switch as its loss leads to apoptosis at 8–14 hpi instead of lysis [32]. This suggests an elegant mechanism for the purpose of 2B* temporal regulation: sufficient levels of 2A are required to induce the ribosomal frameshift necessary for 2B* translation, at which point 2A may subvert the caspase-3 cleavage indirectly induced by 2B* (Figs6  7), promoting GSDME-mediated pyroptosis over apoptosis.

Elucidating the caspase-3-independent lysis pathway, which is only inefficiently utilized by 2B*KO EMCV, is a topic for future work. It is tempting to hypothesize that this pathway is necroptosis, a caspase-independent mechanism mediated by oligomerization and translocation of MLKL to the plasma membrane. Notably, TMEV − which lacks 2B* − is reported to induce necroptosis in some systems [70]. Adherent cells destined for necroptosis initially lose cell-to-cell contact, prior to rounding, swelling and membrane permeabilization [71]. However, these features are more similar to those of the 2B*KO EMCV-infected MEF cells than the WT EMCV-infected cells (Fig. 5), and 2B*KO EMCV does not efficiently use the caspase-3-independent lysis pathway in HeLa cells (Fig. 8). Although this may reflect cell-specific differences, MEF and HeLa cells exhibited similar cytotoxicity profiles (Fig. 4) and virus growth curve patterns (Fig. 3). The simplistic scenario, wherein viruses which lack 2B* access a conserved caspase-independent pathway such as necroptosis, is unlikely.

Instead, it is more likely that caspase-6 (and/or caspase-8) drives caspase-3-independent cell death during WT EMCV infection, for example, via GSDMD- or GSDMC-mediated pyroptosis [40,41, 72, 73] or PANoptosis (see below) [67,74, 75]. Notably, canonical (GSDMD-driven) pyroptosis would require the activation of caspase-1 and the formation of an inflammasome complex. The activation of the NLRP3 inflammasome has been reported during WT EMCV infection and was ascribed to the viroporin 2B [76]. This is thought to be the cause of the increase in IL-1*β*, which occurs in EMCV-infected macrophages [77]. RAW264.7 macrophages were the one tested cell line, which did not completely reproduce the 2B*KO phenotype (Figs3d  4e), suggesting that certain cell lines may utilize canonical pyroptosis despite the synergism between 2B* and caspase-3. It must also be noted that inflammasome activation has been found to be a subsequent effect, rather than a cause, of WT EMCV-induced cell lysis in certain models [62], highlighting the variability across systems.

These hypotheses, however, ignore the potential explanation that GSDME cleavage is still occurring in the absence of caspase-3, a phenomenon reported recently [78]. The presence of cleaved GSDME in WT EMCV-infected caspase-3 KO HeLa cells was not investigated; thus, GSDME-mediated pyroptosis may remain the source of cell lysis. Double-knockout cells (such as those utilized during EV71 lysis studies [44]), or a specific GSDME inhibitor, are required to test this hypothesis. Whilst caspase-7 would be the obvious culprit for caspase-3-independent GSDME cleavage, as caspase-3 and caspase-7 often function interchangeably, early work suggested that caspase-7 cannot cleave GSDME [35]. Caspase-6 is however able to cleave GSDME, albeit inefficiently [66,67].

Indeed, our data appear to be the first direct evidence that caspase-6 has a role in picornavirus-induced cell lysis, although whether this involves the caspase-3-dependent or -independent pathway (or both) is currently unclear (Fig. 10c). Alongside caspase-3 and caspase-7, caspase-6 is classified as an apoptotic effector (or executioner) caspase, despite not being able to carry out apoptosis alone. Caspase-6 induces the cleavage of caspase-8 [79] to form a positive feedback loop, promoting intrinsic apoptosis; increased caspase-8 and caspase-9 cleavage have both been reported in WT EMCV infections [31,33]. Caspase-6 is also able to cleave caspase-3 directly in some models (although the relevance of these to our particular scenario is questionable) [80]. It is therefore highly conceivable that the activation of caspase-6 (and presumably, subsequent caspase-8 activation) promotes the activation of caspase-3, increasing the efficiency of the caspase-3-dependent, GSDME-mediated cell lysis observed during WT EMCV infection.

Intriguingly, caspase-6 and/or caspase-8 may also drive the caspase-3-independent pathway induced during WT EMCV infection, as caspase-8 would presumably cleave GSDMC [40] (and possibly GSDMD [42]), mediating canonical pyroptosis. The relative contributions of caspase-6 and caspase-8 to cell death during WT EMCV infection will be a subject for future work. Regardless of the protease involved, the caspase-3-independent pathway is clearly supported by the presence of 2B*. We have no evidence that the viral protease can directly cleave either caspases or gasdermins, marking a difference between picornaviruses: compare this to the 3C^pro^ of EV71, which cleaves caspase-8 and caspase-9, thus indirectly activating caspase-3 [81]. Coxsackievirus B3 3C^pro^ also induces caspase-8 cleavage and subsequent activation of caspase-3, as well as activating the intrinsic apoptotic pathway and activation of caspase-9 [82]. FMDV 3C^pro^ is notably able to cleave GSDME directly [45], inducing pyroptosis independent of caspase-3 activity.

Whilst it is possible that the cell lysis pathways promoted by 2B* require two entirely separate functions of 2B*, our current hypothesis is that 2B* promotes a shared early stage, which then branches into separate lytic mechanisms at later timepoints. The intertwined nature of cell death pathways, as a result of caspase crosstalk, is being increasingly recognized: the three major cell death pathways – pyroptosis, apoptosis and necroptosis – have such a close relationship that they can even occur simultaneously, resulting in PANoptosis. First shown to occur during influenza A virus infection [74], the PANoptosome multiprotein complex includes components from each of the three cell death pathways, such as NLRP3, RIKP1, RIPK3 and caspase-8, and relies upon caspase-6 [83]. It is clear therefore that whilst we have identified two distinct lytic cell death pathways that operate during EMCV infection, others may contribute during infection *in vivo* or in other model systems. Given the existence of four other gasdermins (some of which can be activated by multiple caspases), it cannot be concluded that the combination of caspase-3 and GSDME is solely responsible for WT EMCV-induced cell lysis, as an alternative caspase:gasdermin combination may contribute.

Due to the parallels that we observed between virus titre and cell lysis (Figs3  4), we did not investigate non-lytic virus release in this study. However, it is well known that picornaviruses manipulate intracellular membranes [84,85] and hijack vesicle release mechanisms in multiple cell lines [86,87], enabling the release of virions through the intact cell membrane prior to lysis [55,60]. It remains possible that 2B* regulates virus non-lytic release, although we suspect that this would have a minor effect on released virus titre in comparison with cell lysis. It must also be noted that although the non-lytic release is reported for many picornaviruses, including enterovirus [59] and poliovirus [58], the process is clearly not wholly conserved across the *Picornaviridae* as, in the case of EMCV, L is known to promote virion-containing vesicle secretion [13] and yet it is not encoded by enteroviruses. It remains possible therefore that 2B* may influence this process specifically during EMCV infection. Were 2B* to affect non-lytic release, it is likely that it would simultaneously affect the autophagic pathways, as EMCV non-lytic release from HeLa cells is promoted by autophagy dysregulation. The EMCV L protein is also known to redirect autophagy, away from protein turnover and towards the secretory pathway, highlighting the links between these two processes. Autophagy is manipulated during picornavirus infection to allow increased viral replication and the formation of replication organelles [57,86, 88]. As disruption of this highly regulated process can lead to cell death, subversion of the autophagic machinery to form replication organelles may be linked to the cell death pathways utilized during WT EMCV infection.

To conclude, we report that WT EMCV induces lytic cell death via two seemingly distinct mechanisms, one caspase-3 dependent (presumably GSDME-mediated pyroptosis) and the other caspase-3 independent. Both of these pathways require 2B* for maximum efficiency, although the mechanisms for this remain undefined. The synergism between 2B* and caspase-3 allows WT EMCV to induce lysis earlier and at a lower threshold of cleaved caspase-3 than 2B*KO EMCV. We present a model of cell death following infection, where either the presence of 2B* or caspase-3 is sufficient to induce cell lysis but the presence of both enables this to occur more rapidly. The delay in lytic cell death in the absence of 2B* explains the small plaque phenotype and poor extracellular virus titres observed during 2B*KO EMCV infection.

## supplementary material

10.1099/jgv.0.002075 Uncited Fig. S1.

## Abbreviations

BDMA: benzyldimethylamine
CPE: cytopathic effect
DMEM: Dulbecco’s Modified Eagle Medium
EMCV: encephalomyocarditis virus
FMDV: foot and mouth disease virus
GCU: green colorimetric unit
hpi: hours post-infection
KO: knockout
MEF: mouse embryonic fibroblast
PRF: programmed ribosomal frameshifting
RLU: relative luciferase unit
STS: staurosporine
TEM: transmission electron microscopy
TMEV: Theiler’s murine encephalomyelitis virus
