# Supplementary material for: The EMCV protein 2B* is required for efficient cell lysis via both caspase-3-dependent and -independent pathways during infection
Source: J Gen Virol. 2025 Feb 10;106(2):002075. doi: 10.1099/jgv.0.002075 (PMC11811419; doi:10.1099/jgv.0.002075)
Supplement: Uncited Fig. S1. [file jgv-106-02075-s001.pdf]

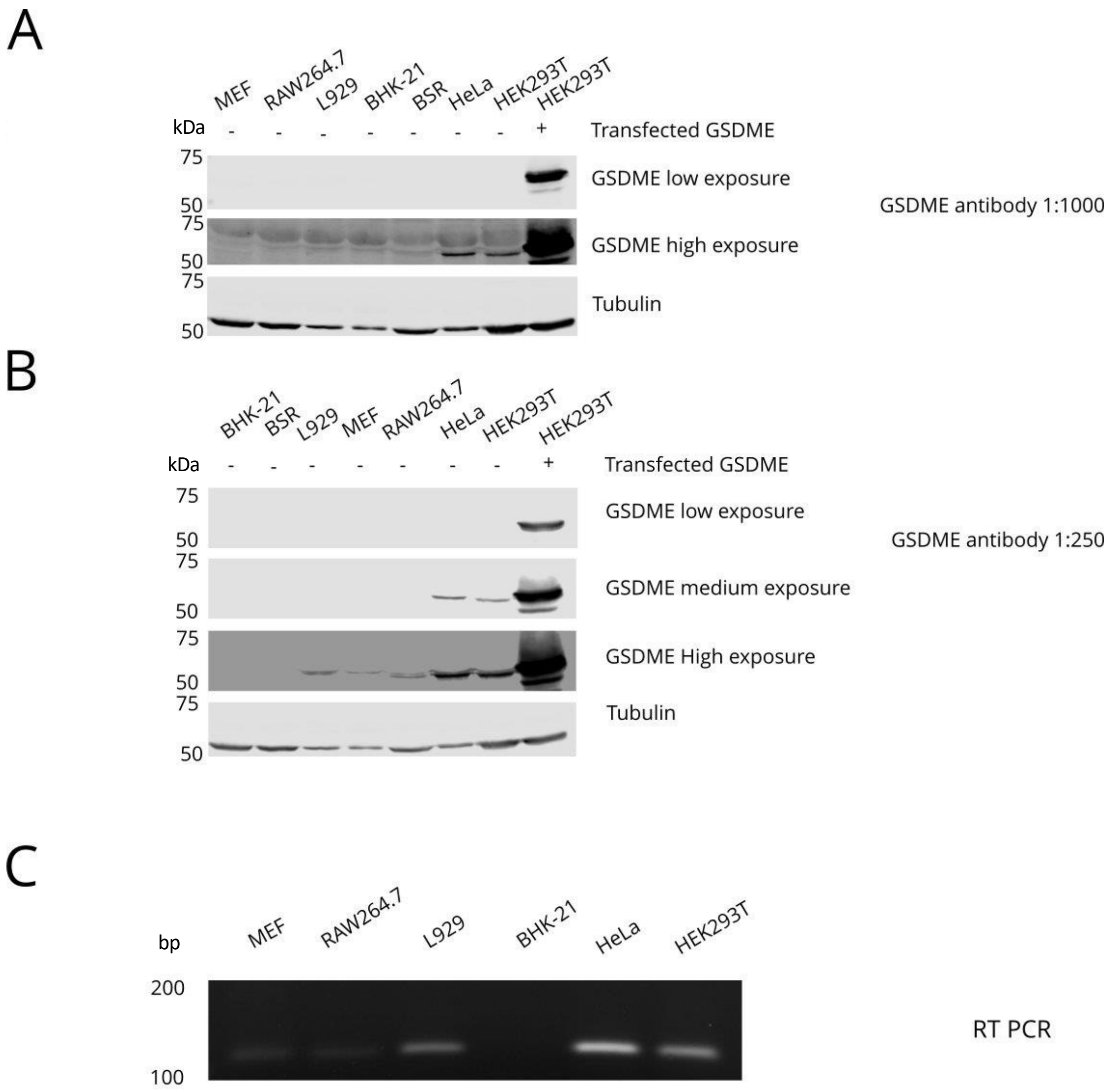

Supplementary Figure 1: Confirmation of GSDME expression in all non-hamster cell lines. GSDME was detected in cell lysates by immunoblot using a commercial antibody at the recommended dilution (A) and a more concentrated dilution (B). As RAW264.7 and L929 cells have been previously reported to express GSDME, they were included as positive controls to ensure the mouse epitope was recognised by the antibody, which was raised against human GSDME. HEK293T cells transiently transfected with pcDNA3.1-GSDME were included as an additional control. GSDME mRNA was detected by RT-PCR (C).
